# Supplementary material for: Evidence for high inter-generational individual quality in yellow-eyed penguins
Source: PeerJ. 2017 Feb 28;5:e2935. doi: 10.7717/peerj.2935 (PMC5333550; doi:10.7717/peerj.2935)
Supplement: Supplemental Information 1 [file peerj-05-2935-s001.docx]

**Supplementary material: High inter-generational individual quality in yellow-eyed penguins**

Stein, A.M., Young, M.J., Darby, J.T., Seddon, P.J., and van Heezik, Y.

All statistical analyses were carried out using R (Version 3.3.1, R Core Team 2016). The data file “LRSSUMMARY.csv” is a summary of the lifetime reproductive success, life-history, offspring and grand-offspring of 130 founding generation sexed yellow-eyed penguins with complete life-histories, as described in the manuscript.

**S1: Statistical analyses**

Wilcoxon Rank-Sum tests were used to test for differences between male and female yellow-eyed penguins for each of the life-history parameters considered (lifetime reproductive success, LRS; Age-at-first-breeding; Lifespan; Breeding lifespan; total number of mates; total number of first-generation offspring to survive juvenile year; total number of first-generation offspring to attempt breeding; total number of first-generation offspring to breed successfully; total number of breeding attempts). The function *wilcox.test* from the package *stats* was used (R Core Team 2016):

> wilcox.test(LRS[SEX=="F"], y = LRS[SEX=="M"], alternative= "two.sided", conf.level=0.95)

> wilcox.test(AGEATFIRSTBREEDING[SEX=="F"], y = AGEATFIRSTBREEDING[SEX=="M"], alternative= "two.sided", conf.level=0.95)

> wilcox.test(LIFESPAN[SEX=="F"], y = LIFESPAN[SEX=="M"], alternative= "two.sided", conf.level=0.95)

> wilcox.test(BREEDINGLIFESPAN[SEX=="F"], y =BREEDINGLIFESPAN[SEX=="M"], alternative= "two.sided", conf.level=0.95)

> wilcox.test(TOTALMATES[SEX=="F"], y = TOTALMATES[SEX=="M"], alternative= "two.sided", conf.level=0.95)

> wilcox.test(SURVIVINGJUVENILES[SEX=="F"], y = SURVIVINGJUVENILES[SEX=="M"], alternative= "two.sided", conf.level=0.95)

> wilcox.test(BREEDINGOFFSPRING[SEX=="F"], y = BREEDINGOFFSPRING[SEX=="M"], alternative= "two.sided", conf.level=0.95)

> wilcox.test(SUCCESSFULBREEDINGOFFSPRING[SEX=="F"], y = SUCCESSFULBREEDINGOFFSPRING[SEX=="M"], alternative= "two.sided", conf.level=0.95)

> wilcox.test(TOTALNOBREEDINGATTEMPTS[SEX=="F"], y = TOTALNOBREEDINGATTEMPTS[SEX=="M"], alternative= "two.sided", conf.level=0.95)

**S2: Consideration of cohort as a predictor of LRS**

We did not include ‘Cohort’ as a factor in the models identifying determinants of LRS. Longer-lived birds in later cohorts (1994-2003) had not completed their breeding lifetimes and a result were not included in our study, whereas shorter-lived birds that were marked from 1994 to 2003 that had died by 2007 were included. If we had included ‘cohort’ in our analyses it would have given a false impression that the super breeder phenomenon ceased at 1994. Many long-lived birds are in fact still alive and breeding at Boulder Beach in the 2016/17 season (oldest identified is 23 years old).

We ran a generalised linear model (GLM) on a subset of the total data set, which included birds from cohorts 1981 – 1994 for which we had complete lifespans (n = 161). The life-history response variables (lifespan, age-at-first-breeding, total mates, sex, and the appropriate interaction terms) as well as cohort were included in the full model. All derivatives of this model were assessed using QAICc and the top 2ΔQAICc models were model-averaged (after Burnham and Anderson 2002), with the model-averaged results of the best five models being presented in Table S2.

Our results indicate that cohort was included in all five best models and therefore had high relative importance, however only one year out of 14 cohort years had a significant negative effect relative to other effect sizes, and in the remaining 13 cohort years the standard errors were as large as the effect sizes (Table S2). We concluded from this analysis that there was limited compelling evidence to include cohort in further modelling of lifetime reproductive success.

**Table S2.** Model-averaged generalised linear model of lifetime reproductive success (LRS) and life-history parameters of 161 yellow-eyed penguins that were marked at Boulder Beach, New Zealand between 1981 and 1994 (all non-binary data are standardized to have mean = 0 and SD = 1).

|  | Estimate | SE | 95% Confidence Interval | Relative Importance |
| --- | --- | --- | --- | --- |
| (Intercept)* | 1.60 | 0.15 | (1.30, 1.90) | - |
| factor(COHORT)1982 | -0.57 | 0.21 | (-0.98, -0.16) | 1.00 |
| factor(COHORT)1983 | -0.19 | 0.18 | (-0.55, 0.17) | 1.00 |
| factor(COHORT)1984 | 0.33 | 0.25 | (-0.16, 0.82) | 1.00 |
| factor(COHORT)1986 | 0.26 | 0.50 | (-0.72, 1.24) | 1.00 |
| factor(COHORT)1987 | 0.20 | 0.18 | (-0.16, 0.56) | 1.00 |
| factor(COHORT)1988 | 0.28 | 0.17 | (-0.06, 0.61) | 1.00 |
| factor(COHORT)1989 | 0.15 | 0.25 | (-0.35, 0.65) | 1.00 |
| factor(COHORT)1990 | 0.07 | 0.46 | (-0.83, 0.97) | 1.00 |
| factor(COHORT)1991 | 0.24 | 0.22 | (-0.20, 0.67) | 1.00 |
| factor(COHORT)1992 | 0.20 | 0.18 | (-0.16, 0.56) | 1.00 |
| factor(COHORT)1993 | 0.32 | 0.18 | (-0.03, 0.67) | 1.00 |
| factor(COHORT)1994 | -0.12 | 0.20 | (-0.52, 0.27) | 1.00 |
| factor(SEX)M ^ⱡ^ | -0.14 | 0.09 | (-0.31, -0.01) | 0.85 |
| zAGEATFIRSTBREEDING | -0.05 | 0.05 | (-0.16, 0.01) | 0.61 |
| zLIFESPAN ^ⱡ^ | 0.67 | 0.06 | (0.55, 0.78) | 1.00 |
| zTOTALMATES | -0.06 | 0.05 | (-0.17, 0.00) | 0.73 |

*Sex (Female) is the reference category

*factor(COHORT)1981 is the reference category

^ⱡ^ Significant results

Full model statement: glm(LRS ~ zLIFESPAN + zAGEATFIRSTBREEDING + zTOTALMATES + factor(SEX) + factor(COHORT) + factor(SEX):zAGEATFIRSTBREEDING + factor(SEX):zLIFESPAN

**S3: Model selection tables**

Generalised linear mixed models (GLMM) were used to construct a maximal model to explain the variation in lifetime reproductive success of yellow-eyed penguins, using life-history parameters and sex as predictors. To control for the pseudoreplication introduced by mated pairs being included in the analysis, the random factor “mate code” was used in the models. The life-history data for each bird remained in summary form, however for each year of breeding the mate code varied to account for different mates. All life-history parameters were z-transformed using the function *scale* from the package *base* (R Core Team 2016). We used a Poisson distribution, using a function to return log likelihoods for QAICc. Due to model selection uncertainty, we used model averaging across the best 2ΔQAICc models. We used the functions *dredge*, *get.models*, *model.avg* from the package *MuMIn* (Bartoń 2015), the function *r.squaredGLMM* from the package *AICcmodavg* was used to determine maximal model fit (Mazerolle 2016), and the function *confint* from the package *stats* (R Core Team 2016) to simplify model selection and multi-model inference with our *a priori* selected input variables:

> GLMM1<-glmer(LRS ~ zLIFESPAN + zAGEATFIRSTBREEDING + zTOTALMATES + factor(SEX) + factor(SEX):zAGEATFIRSTBREEDING + factor(SEX):zLIFESPAN + (1|MATECODE), data=Data, family= poisson, glmerControl(optimizer="bobyqa", optCtrl=list(maxfun=20000)))

> r.squaredGLMM(GLMM1)

> chat<-sum(residuals(GLMM1, "pearson")^2)/GLMM1$df.residual

> options(na.action = "na.fail")

> DREDGEDMODELSET<-dredge(GLMM1, beta= "none", evaluate = TRUE, rank = QAICc, chat=chat)

> options(na.action = "na.omit")

> BESTMODELS<-get.models(DREDGEDMODELSET, subset = delta<2)

> BESTMODELSET<-model.avg(BESTMODELS)

> summary(BESTMODELSET)

> confint(BESTMODELSET)

| **Model statement** | **df** | **logLik** | **QAICc** | **ΔQAICc** | **Akaike weight** |
| --- | --- | --- | --- | --- | --- |
| LRS ~ factor(SEX) + zAGEATFIRSTBREEDING + zLIFESPAN + factor(SEX):zAGEATFIRSTBREEDING | 6 | -1865.54 | 3743.19 | 0.00 | 0.35 |
| LRS ~ factor(SEX) + zAGEATFIRSTBREEDING + zLIFESPAN + factor(SEX):zAGEATFIRSTBREEDING + factor(SEX):zLIFESPAN | 7 | -1865.32 | 3744.78 | 1.59 | 0.16 |
| LRS ~ factor(SEX) + zAGEATFIRSTBREEDING + zLIFESPAN + zTOTALMATES + factor(SEX):zAGEATFIRSTBREEDING | 7 | -1865.37 | 3744.87 | 1.68 | 0.15 |
| LRS ~ factor(SEX) + zAGEATFIRSTBREEDING + zLIFESPAN | 5 | -1867.86 | 3745.80 | 2.60 | 0.10 |
| LRS ~ factor(SEX) + zAGEATFIRSTBREEDING + zLIFESPAN + factor(SEX):zLIFESPAN | 6 | -1866.86 | 3745.82 | 2.63 | 0.09 |
| LRS ~ factor(SEX) + zAGEATFIRSTBREEDING + zLIFESPAN + zTOTALMATES + factor(SEX):zAGEATFIRSTBREEDING + factor(SEX):zLIFESPAN | 8 | -1865.16 | 3746.49 | 3.30 | 0.07 |
| LRS ~ factor(SEX) + zAGEATFIRSTBREEDING + zLIFESPAN + zTOTALMATES | 6 | -1867.77 | 3747.65 | 4.46 | 0.04 |
| LRS ~ factor(SEX) + zAGEATFIRSTBREEDING + zLIFESPAN + zTOTALMATES + factor(SEX):zLIFESPAN | 7 | -1866.77 | 3747.68 | 4.49 | 0.04 |
| LRS ~ factor(SEX) + zLIFESPAN + factor(SEX):zLIFESPAN | 5 | -1871.95 | 3753.98 | 10.78 | 0.00 |
| LRS ~ factor(SEX) + zLIFESPAN + zTOTALMATES + factor(SEX):zLIFESPAN | 6 | -1871.93 | 3755.97 | 12.78 | 0.00 |
| LRS ~ zAGEATFIRSTBREEDING + zLIFESPAN | 4 | -1874.17 | 3756.39 | 13.20 | 0.00 |
| LRS ~ factor(SEX) + zLIFESPAN | 4 | -1874.22 | 3756.49 | 13.30 | 0.00 |
| LRS ~ zAGEATFIRSTBREEDING + zLIFESPAN + zTOTALMATES | 5 | -1874.16 | 3758.38 | 15.19 | 0.00 |
| LRS ~ factor(SEX) + zLIFESPAN + zTOTALMATES | 5 | -1874.18 | 3758.44 | 15.25 | 0.00 |
| LRS ~ zLIFESPAN | 3 | -1880.59 | 3767.22 | 24.03 | 0.00 |
| LRS ~ zLIFESPAN + zTOTALMATES | 4 | -1880.45 | 3768.96 | 25.76 | 0.00 |
| LRS ~ zAGEATFIRSTBREEDING + zTOTALMATES | 4 | -1990.51 | 3989.06 | 245.87 | 0.00 |
| LRS ~ factor(SEX) + zAGEATFIRSTBREEDING + zTOTALMATES | 5 | -1989.67 | 3989.41 | 246.22 | 0.00 |
| LRS ~ factor(SEX) + zAGEATFIRSTBREEDING + zTOTALMATESfactor(SEX):zAGEATFIRSTBREEDING | 6 | -1989.65 | 3991.41 | 248.22 | 0.00 |
| LRS ~ zTOTALMATES | 3 | -1993.09 | 3992.21 | 249.02 | 0.00 |
| LRS ~ factor(SEX) + zTOTALMATES | 4 | -1992.29 | 3992.63 | 249.44 | 0.00 |
| LRS ~ factor(SEX)+ zAGEATFIRSTBREEDING + factor(SEX):zAGEATFIRSTBREEDING | 5 | -2060.62 | 4131.32 | 388.13 | 0.00 |
| LRS ~ factor(SEX) + zAGEATFIRSTBREEDING | 4 | -2062.43 | 4132.91 | 389.72 | 0.00 |
| LRS ~ zAGEATFIRSTBREEDING | 3 | -2065.83 | 4137.70 | 394.51 | 0.00 |
| LRS ~ factor(SEX) | 3 | -2066.67 | 4139.36 | 396.17 | 0.00 |
| LRS ~ 1 | 2 | -2070.14 | 4144.29 | 401.10 | 0.00 |

A similar approach was used to explain the variation in breeder type as described above, with the maximal model being of the form:

> GLMM2<- glmer(LRS ~ zLIFESPAN + zAGEATFIRSTBREEDING + zTOTALMATES + factor(BREEDERTYPE) + factor(BREEDERTYPE):zAGEATFIRSTBREEDING + factor(BREEDERTYPE):zLIFESPAN + (1|MATECODE), data=Data, family= poisson, glmerControl(optimizer="bobyqa", optCtrl=list(maxfun=20000)))

> r.squaredGLMM(GLMM2)

> chat<-sum(residuals(GLMM2, "pearson")^2)/GLMM1$df.residual

> options(na.action = "na.fail")

> DREDGEDMODELSET<-dredge(GLMM2, beta= "none", evaluate = TRUE, rank = QAICc, chat=chat)

> options(na.action = "na.omit")

> BESTMODELS<-get.models(DREDGEDMODELSET, subset = delta<2)

> BESTMODELSET<-model.avg(BESTMODELS)

> summary(BESTMODELSET)

> confint(BESTMODELSET)

| **Model statement** | **df** | **logLik** | **QAICc** | **ΔQAICc** | **Akaike weight** |
| --- | --- | --- | --- | --- | --- |
| factor(BREEDERTYPE) + zAGEATFIRSTBREEDING | 6 | -1850.66 | 3713.42 | 0.00 | 0.49 |
| factor(BREEDERTYPE) + zAGEATFIRSTBREEDING | 7 | -1850.32 | 3714.77 | 1.35 | 0.25 |
| factor(BREEDERTYPE) + zAGEATFIRSTBREEDING | 7 | -1850.66 | 3715.46 | 2.03 | 0.18 |
| factor(BREEDERTYPE) + zAGEATFIRSTBREEDING | 8 | -1850.32 | 3716.81 | 3.39 | 0.09 |
| factor(BREEDERTYPE) + zLIFESPAN + zTOTALMATES + factor(BREEDERTYPE):zLIFESPAN | 6 | -1857.51 | 3727.12 | 13.70 | 0.00 |
| factor(BREEDERTYPE) + zLIFESPAN + factor(BREEDERTYPE):zLIFESPAN | 5 | -1858.80 | 3727.68 | 14.25 | 0.00 |
| factor(BREEDERTYPE) + zAGEATFIRSTBREEDING + zLIFESPAN | 5 | -1859.27 | 3728.62 | 15.20 | 0.00 |
| factor(BREEDERTYPE) + zAGEATFIRSTBREEDING + zLIFESPAN + zTOTALMATES | 6 | -1858.67 | 3729.45 | 16.02 | 0.00 |
| factor(BREEDERTYPE) + zAGEATFIRSTBREEDING + zLIFESPAN | 6 | -1859.02 | 3730.14 | 16.72 | 0.00 |
| factor(BREEDERTYPE) + zAGEATFIRSTBREEDING + zLIFESPAN + zTOTALMATES + factor(BREEDERTYPE):zAGEATFIRSTBREEDING | 7 | -1858.37 | 3730.88 | 17.46 | 0.00 |
| factor(BREEDERTYPE) + zLIFESPAN + zTOTALMATES | 5 | -1864.06 | 3738.20 | 24.78 | 0.00 |
| factor(BREEDERTYPE) + zLIFESPAN | 4 | -1865.65 | 3739.36 | 25.94 | 0.00 |
| zAGEATFIRSTBREEDING + zLIFESPAN | 4 | -1874.17 | 3756.39 | 42.97 | 0.00 |
| zAGEATFIRSTBREEDING + zLIFESPAN + zTOTALMATES | 5 | -1874.16 | 3758.38 | 44.96 | 0.00 |
| zLIFESPAN | 3 | -1880.59 | 3767.22 | 53.80 | 0.00 |
| zLIFESPAN + zTOTALMATES | 4 | -1880.45 | 3768.96 | 55.53 | 0.00 |
| factor(BREEDERTYPE) + zAGEATFIRSTBREEDING | 6 | -1930.82 | 3873.74 | 160.32 | 0.00 |
| factor(BREEDERTYPE) + zTOTALMATES | 4 | -1932.88 | 3873.80 | 160.38 | 0.00 |
| factor(BREEDERTYPE) + zAGEATFIRSTBREEDING | 5 | -1932.18 | 3874.44 | 161.02 | 0.00 |
| zAGEATFIRSTBREEDING + zTOTALMATES | 4 | -1990.51 | 3989.06 | 275.64 | 0.00 |
| zTOTALMATES | 3 | -1993.09 | 3992.21 | 278.79 | 0.00 |
| factor(BREEDERTYPE)+ zAGEATFIRSTBREEDING | 4 | -1999.58 | 4007.21 | 293.78 | 0.00 |
| factor(BREEDERTYPE) | 3 | -2000.72 | 4007.47 | 294.05 | 0.00 |
| factor(BREEDERTYPE)+ zAGEATFIRSTBREEDING + factor(BREEDERTYPE):zAGEATFIRSTBREEDING | 5 | -1999.02 | 4008.11 | 294.68 | 0.00 |
| zAGEATFIRSTBREEDING | 3 | -2065.83 | 4137.70 | 424.28 | 0.00 |

**References**

Bartoń K, 2016. MuMIn: Multi-Model Inference. R package version 1.15.6. https://CRAN.R-project.org/package=MuMIn.

Burnham KP, and Anderson DR, 2002. Model selection and multimodel inference: A practical

information-theoretic approach. Second edition. Springer-Verlag, New York USA. 488p.

Mazerolle MJ, 2016. AICcmodavg: Model selection and multimodel inference based on (Q)AIC(c). R package version 2.0-4. http://CRAN.R-project.org/package=AICcmodavg.

R Core Team, 2016. R: A language and environment for statistical computing. R Foundation for Statistical Computing, Vienna, Austria. URL https://www.R-project.org/.

R for Fish and Wildlife Grads, 2016. Accessed 10 August 2016. Website: https://sites.goo
